# Cognitive Impairment and Endoplasmic Reticulum Stress Induced by Repeated Short-Term Sevoflurane Exposure in Early Life of Rats

**DOI:** 10.3389/fpsyt.2018.00332

**Published:** 2018-08-02

**Authors:** Fu-Yi Shen, Ying-Cai Song, Fei Guo, Zhen-Dong Xu, Qian Li, Bing Zhang, Yu-Qin Ma, Yue-Qi Zhang, Rong Lin, Yang Li, Zhi-Qiang Liu

**Affiliations:** ^1^Department of Anesthesiology, Shanghai First Maternity and Infant Hospital, Tongji University School of Medicine, Shanghai, China; ^2^Key Laboratory of Receptor Research, Shanghai Institute of Materia Medica, Chinese Academy of Sciences, Shanghai, China; ^3^University of Chinese Academy of Sciences, Beijing, China

**Keywords:** sevoflurane, repeated exposure, cognitive dysfunction, synaptic plasticity, endoplasmic reticulum stress

## Abstract

Sevoflurane is one of the most commonly used volatile anaesthetics for children, but the safety of prolonged or repeated clinical use of sevoflurane in infants or children is controversial. Here, we investigated the effects of sevoflurane on rats in early life and the time scale of those effects. Our behavioral results indicated that repeated short-term exposure of new-born rats to sevoflurane caused learning and memory impairment, while a single exposure of rats to sevoflurane was relatively safe. Further mechanistic investigation revealed that repeated sevoflurane exposure impaired long-term potentiation (LTP), downregulated the expression of certain synaptogenesis-related proteins (GluR1, PSD95) and upregulated proteins related to endoplasmic reticulum (ER) stress in the hippocampus. An ER stress inhibitor, tauroursodeoxycholic acid (TUDCA), reversed the changes in the levels of synaptic plasticity proteins. Our results provide new evidence for the clinical concerns regarding repeated sevoflurane anesthesia.

## Introduction

Sevoflurane, a volatile anesthetic, is commonly used in pediatric anesthesia, owing to its rapid onset, short recovery time, sweet smell and nonflammability. Sevoflurane has been used clinically for decades, and most studies have supported its safety for adults. However, studies have shown that sevoflurane causes stress and neurotoxicity in the developing brains of rodents and non-human primates (NHPs) (1, 2). In addition, a large number of clinical observations have demonstrated that childhood exposure to anesthesia can cause long-term cognitive impairment (3, 4). Since the developing brain is susceptible to anesthetics (5, 6), the US Food & Drug Administration (FDA) has also raised concerns regarding the effects of repeated or prolonged anesthetic exposure in children (7).

As the developing brain is highly sensitive to different environmental factors such as sensory stimuli, drugs and stress, children's brains can be at high risk of being remodeled by severe anesthetic exposure. Since sevoflurane is one of the most commonly used volatile anesthetics for children, its safety deserves further investigation. Many reports have confirmed that a single exposure to sevoflurane has no effects on the infants or children (8). However, some research has reported long-term cognitive impairment caused by repeated sevoflurane exposure (2, 9–11). Unfortunately, some major surgeries in clinical practice require repeated sevoflurane exposure for infants or children; therefore, it is urgent to clarify the effects of repeated sevoflurane anesthesia and its long-term effects later in life.

In the present study, we investigated the time scope of sevoflurane exposure in neonatal rats by comparing single and repeated exposure to sevoflurane. Our results indicate that repeated exposure of young rats to sevoflurane results in severe deterioration of memory and cognition later in life, while a single sevoflurane exposure is relatively safe. Furthermore, the mechanism underlying the adverse effects of repeated sevoflurane exposure may involve dysfunction of synaptic plasticity and endoplasmic reticulum (ER) stress.

## Materials and methods

### Animals

**Figure 1:**
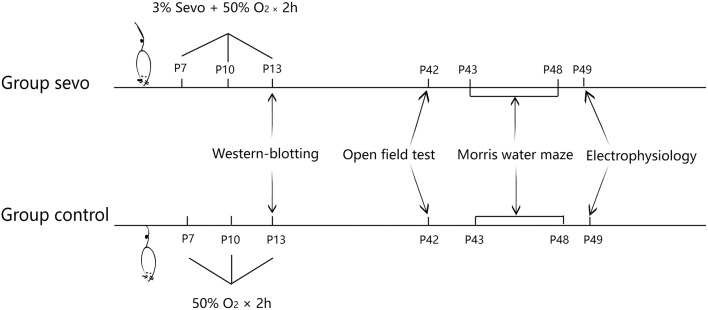
Schematic timeline of the experimental procedure.

The present study was approved by the Animal Care Committees of Shanghai Institute of Materia Medica, Chinese Academy of Science, and the experiments were carried out in accordance with EU Directive 2010/63/EU on the protection of animals used for scientific purposes. Four-day-old male Sprague Dawley (SD) rats obtained from Shanghai Sippr-BK Laboratory Animal Co were housed with their dams under controlled illumination (12-h/12-h light/dark cycle, light from 07:00 to 19:00) at 24 ± 1°C. The rats were given free access to food and water. On postnatal day (P) 7, all neonatal rats were randomly divided into 2 groups: a control group and a sevoflurane (sevo) group. The neonatal rats in the sevo group were exposed to 3% sevoflurane for 2 h per exposure on P7, P10, and P13, whereas those in the control group were subjected to the same conditions without receiving sevoflurane. Changes in synaptic plasticity and ER-stress-associated protein levels were then analyzed by western blotting (*n* = 4 for each group). On P21, all the young rats were weaned and housed in groups of 2 per cage in standard conditions. Behavioral tests were later conducted: the open field test for locomotor activity was performed on P42, and the Morris water maze test for learning and memory ability was performed from P43 to P48 (control group: *n* = 10, sevo group: *n* = 9). Subsequently, rats that had undergone behavioral tests were subjected to field excitatory postsynaptic potential (fEPSP) experiments to detect whether synaptic transmission was affected (Figure 1, *n* = 6 for each group).

### Anesthesia

The neonatal rats in the sevo group received 3% sevoflurane in 50% O_2_/N_2_ for 2 h on P7, 10, and 13 in a sealed box at a set temperature of 37 ± 1°C. The rats in the control group received 50% O_2_/N_2_ at the same flow rate in a similar box on the same days. The neonatal rats breathed spontaneously. The concentrations of sevoflurane and oxygen were measured continuously (Vamos, Drager, Germany). Following sevoflurane exposure, the neonatal rats were returned to their dams after recovering their righting reflex.

### Drug treatment

Tauroursodeoxycholic acid (TUDCA, sodium salt; Sigma-Aldrich) was dissolved in 0.9% saline at a concentration of 50 mg/ml and was intraperitoneally injected 1 h before each sevoflurane exposure at a dosage of 50 mg/kg body weight. The rats were randomly divided into four groups: the saline-treated control group (control), the TUDCA-treated control group (TUDCA), the saline-treated sevo group (sevo) and the TUDCA-treated sevo group (sevo+TUDCA).

### Western-blotting analysis

Rats were deeply anesthetized with an injection of chloral hydrate (400 mg/kg, intraperitoneally). Immediately after decapitation, the hippocampus was dissected and homogenized by sonic disruption. The homogenate was centrifuged at 14,000 rpm for 10 min at 4°C. Total protein concentrations were measured using a BCA assay (Pierce, Rockford, IL). The proteins were separated on SDS-PAGE gels and transferred to nitrocellulose membranes. The membranes were blocked and then incubated overnight at 4? with primary antibodies (anti-GRP 78, 1:1,000, Sigma-Aldrich; anti-PERK, 1:1,000, Sigma-Aldrich; anti-eIF-2α, 1:1,000, Sigma-Aldrich; GluR1 subunit (pSer845), 1:1,000, Sigma-Aldrich; anti-PSD95, 1:1,000, Sigma-Aldrich; anti-CREB, 1:1,000, Sigma-Aldrich; anti-GAPDH, 1:1,000, Sigma-Aldrich). Band intensities were measured using Image Processing and Analysis in Java (ImageJ) software.

### Open field test

The open field test is the standard way to obtain an overview of locomotor activity. We performed this test on P42 according to a previously described protocol (12, 13). First, the SD rats were allowed to habituate to the testing room. Subsequently, they were placed in the middle of a square box (100 × 100 × 45 cm) made of black acrylic plastic. The rats were permitted to explore in the box for 10 min, and their movements were video tracked. The total distance moved was calculated offline with an analysis-management system (Viewer 2 Tracking Software, Ji Liang Instruments, China).

### Morris water maze

The Morris water maze test was conducted 24 h after the open field test. The Morris water maze test consisted of 5 days of place navigation training and a probe trial on day 6. The maze was composed of a circular black metal tank (180 cm in diameter, 60 cm in height) filled with water (24 ± 2°C); a video camera set in the ceiling and was connected to a computer equipped with an analysis-management system (Viewer 2 Tracking Software, Ji Liang Instruments, China). The maze was divided into 4 equal quadrants, one of which contained a hidden escape platform (10 cm diameter) in the middle, 0.5 cm below the surface of the water. White marks were located equidistantly around the edge of the maze in all start positions. On P43, each rat was allowed to swim freely in the pool for 60 s for habituation. From P44 to P47, a hidden escape platform (10 cm in diameter) was submerged 0.5 cm below the surface of the water in the middle of the target quadrant. During navigation training, four trials separated by 30 s were conducted daily for each rat. Each day, rats were placed into four different starting quadrants, and entry from the north, south, east, or west point was varied in a quasi-random order. Each rat was allowed a maximum of 60 s to locate the platform. If the rat did not find the platform within 60 s, it was guided to the location. The time that the rat took to reach the submerged platform (escape latency) was recorded to assess spatial learning ability. On P48, a probe test consisting of a 60 s trial with the platform removed was conducted to assess memory. The amount of time spent in the target quadrant was recorded. The target quadrant was defined as the quadrant that previously contained the platform, whose radius was limited to 16 cm in this assessment.

### Slice preparation

SD rats at P49-P55 were deeply anesthetized with an injection of chloral hydrate (400 mg/kg, intraperitoneally) and euthanized by decapitation. The brain tissue was obtained immediately. The hippocampus was cut into 400-μm-thick sections using a vibratome (Leica VT1000 S, USA) in ice-cold, oxygenated modified artificial cerebrospinal fluid (mACSF, in mM: 25.0 NaHCO_3_, 1.25 NaH_2_PO_4_, 2.5 KCl, 0.5 CaCl_2_, 7.0 MgCl_2_, 25.0 glucose, 11.0 choline chloride, 11.6 ascorbic acid and 3.1 pyruvic acid, gassed with 5% CO_2_ and 95% O_2_). The cut slices were quickly placed into a chamber and incubated in normal oxygenated ACSF (118 NaCl, 2.5 KCl, 26 NaHCO_3_, 1 NaH_2_PO_4_, 10 glucose, 1.3 MgCl_2_ and 2.5 CaCl_2_ in mM, gassed with 5% CO_2_ and 95% O_2_) at 32°C for 1 h. Finally, the slice was transferred to the recording chamber at room temperature.

### Electrophysiology

We recorded fEPSPs by using a Multiclamp 700B amplifier (Molecular Devices, USA) under a microscope (Olympus, Japan). For fEPSP recording, both bipolar stimulating and recording electrodes were placed at CA3/Schaffer collateral-CA1 synapses in the stratum radiatum of the CA1 area. The data were filtered at 2 kHz, sampled at 10 kHz using a Digidata 1440A (Molecular Devices, USA) and analyzed with Clampfit 10.2 software (Molecular Devices, USA). The stimuli consisted of 100-μs pulses delivered at 0.03 Hz using an electronic stimulator (Nihon Kohden Corporation, Japan), and the stimulus intensity was adjusted to 65% of the maximal response. The evoked response was monitored for 10 min at this intensity to ensure stable recording prior to the drug treatment. After 10-min stable recording at baseline, the long-term potentiation (LTP) was induced by application of the high frequency intensity (HFS) protocol consisting of 1 s trains of 100 Hz stimulation repeated two times 20 s apart. All recordings were continued for 60–80 min.

### Statistical analysis

The data are expressed as the means ± SEM. The software Statistical Package for the Social Sciences (SPSS, Version 19.0; SPSS Inc., Chicago, IL, USA) was used for statistical analysis. The results of the open field test, and western blots in SD rats were analyzed using unpaired *t*-tests. The results of Morris water maze test were analyzed using a repeated ANOVA in which treatment was used as the between factor and time (weeks) was used as the within factor, and a *post-hoc* LSD test was performed. *p*-values < 0.05 were considered statistically significant.

## Results

### Early-life repeated sevoflurane exposure led to long-term cognitive impairment

**Figure 2:**
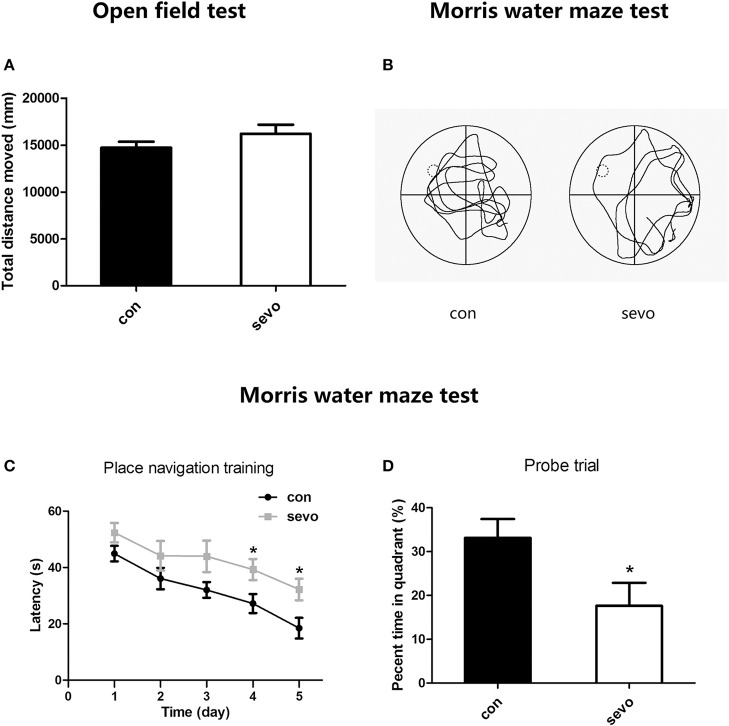
Effects of repeated early-life sevoflurane exposure on behavior. **(A)** In the open field test, no significant difference was observed between the control group and the sevoflurane group in total distance moved. **(B)** A typical path during the probe trial for each group. **(C)** The latency to find the platform during the place navigation training phase of the Morris water maze test. **(D)** The percentage of time spent in the target quadrant during the probe trial of the Morris water maze test. *N* = 10 rats for each group. Repeated ANOVA with a *post hoc* least significant difference (LSD) test and unpaired *t*-tests were performed for **(A,C,D)**, respectively; ^*^p < 0.05. Data are presented as the mean ± SEM.

We first performed the open field test to detect differences in locomotor activity between groups. The results showed that there was no significant difference in total distance moved between the control group and the sevoflurane group, indicating that repeated early-life sevoflurane exposure did not affect the psychomotor ability of animals (Figure 2A).

To examine the long-term effects of early sevoflurane exposure on cognitive function, we administered the Morris water maze test from P43 to P48, consisting 5 days of place navigation training to evaluate learning ability and 1 day (P48) containing a probe trial to assess memory ability. The results suggested that the latency to locate the hidden platform was significantly longer in the sevoflurane group than in the control group on days 4-5 (P46-47) of navigation training, indicating much lower learning ability in sevoflurane-treated animals (Figure 2C). Moreover, the probe trial results showed that the percentage of time spent in the target quadrant was higher for the control group than for the sevoflurane group (Figure 2D). Taken together, all those results imply that repeated early-life sevoflurane exposure caused long-term dysfunction of learning and memory.

### Repeated short-term sevoflurane exposure caused dysfunction of synaptic plasticity in the hippocampus

**Figure 3:**
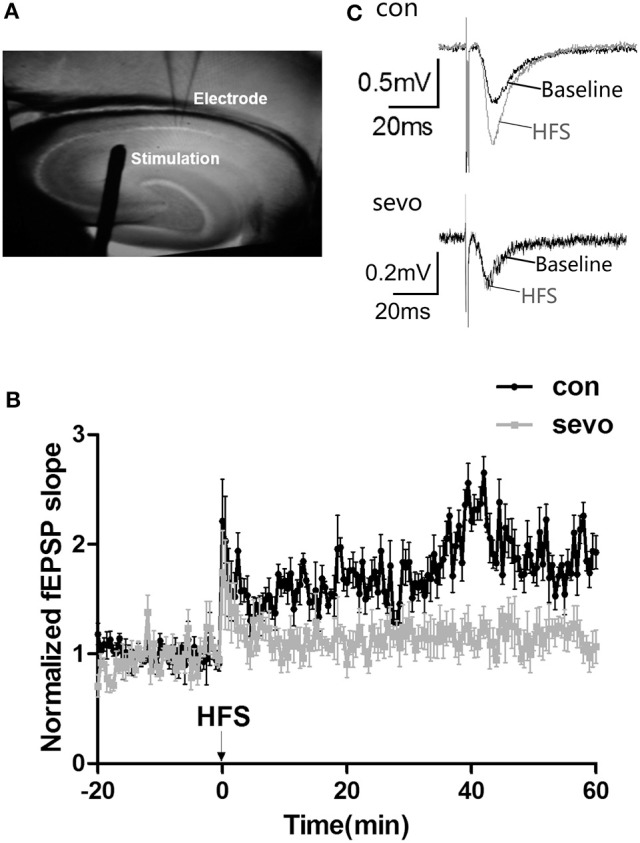
Repeated sevoflurane exposure abolished LTP in the hippocampus. **(A)** Schematic representation of bipolar stimulating and recording electrode placement. **(B)** Tetanic stimulation (one bout of stimulation at 100 Hz) induced LTP in the Schaffer collateral-CA1 pathway of rat hippocampal slices. **(C)** Representative traces of the fEPSP waves were recorded at baseline (the black line) and after repeated sevoflurane exposure (the gray line); early-life sevoflurane exposure impaired LTP in the rat hippocampus. *N* = 9 slices, 4 rats for the control group; *n* = 8 slices, 4 rats for the sevo group.

Sevoflurane exposure may cause an impairment of hippocampal synaptic plasticity, as a severe deficiency of learning and memory ability was observed in the above results (as shown in Figure 2). We then examined the LTP at CA3/Schaffer collateral-CA1 synapses of behaviorally tested rats. Our results showed that the slopes of fEPSPs were significantly decreased in the CA1 area of the hippocampus in the sevoflurane group compared to the control group (Figure 3). This result suggests that early-life repeated sevoflurane exposure abolished LTP, indicating a dysfunction of synaptic plasticity in the hippocampus.

### Expression of proteins associated with synaptic plasticity decreased in the sevoflurane-exposed hippocampus

**Figure 4:**
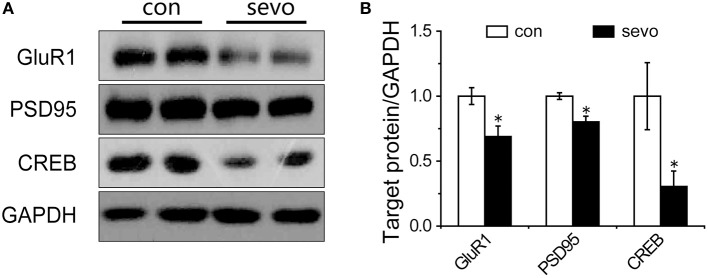
Repeated sevoflurane exposure decreased the expression of GluR1, PSD95, and CREB. **(A)** Representative samples showing the hippocampal expression levels of GluR1, PSD95, and CREB in the control group and the sevoflurane group. **(B)** Densitometric analysis of GluR1, PSD95, and CREB. *N* = 4–6 times for each protein. Unpaired *t*-tests; ^*^p < 0.05 vs. control. Data are presented as the means ± SEM.

To explore possible mechanisms relating to cognitive dysfunction caused by early-life repeated sevoflurane exposure, we further determined the expression levels of synaptic-plasticity-associated proteins under repeated sevoflurane exposure. We examined the expression levels of GluR1, postsynaptic density protein 95 (PSD95) and cAMP response element-binding protein (CREB) in the hippocampus. The results showed that the expression levels of GluR1, PSD95, and CREB were significantly decreased by repeated sevoflurane exposure (Figures 4A,B). Interestingly, the expression levels of the abovementioned proteins did not change with a single sevoflurane exposure (Supplemental Figures S1A,B). These results suggest that repeated sevoflurane exposure but not single exposure decreased hippocampal synaptic plasticity.

### Levels of ER-stress-related proteins in the hippocampus were elevated by repeated sevoflurane exposure

**Figure 5:**
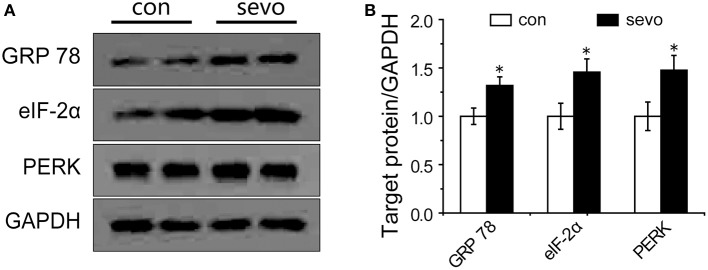
Repeated sevoflurane exposure increased the expression of GRP 78, PERK, and eIF-2α. **(A)** Representative samples showing the expression levels of GRP 78, PERK, and eIF-2α in the hippocampi of the control group and the sevoflurane group; **(B)** Densitometric analysis of GRP 78, PERK, and eIF-2α. *N* = 4–6 times for each protein. Unpaired *t*-tests; ^*^p < 0.05 vs. control. Data are presented as the means ± SEM.

ER stress commonly occurs under severe conditions, such as prolonged anesthetic exposure. It is possible that ER stress might play a critical role in deficits of synaptic plasticity (14). To determine whether sevoflurane exposure was associated with ER stress in the hippocampus, we analyzed the levels of ER-stress-associated proteins in the hippocampus. The results showed that the expression level of glucose-regulated protein 78 (GRP 78), a crucial biomarker of ER stress (15), was significantly elevated by sevoflurane exposure (Figures 5A,B). PKR-like ER kinase (PERK) is a critical transmembrane ER signaling protein that mediates an important signaling pathway when dissociated from GRP 78, and eukaryotic initiation factor 2α (eIF-2α) is a critical protein in the PERK pathway (16, 17). The levels of PERK and eIF-2α were also significantly elevated in the sevoflurane group compared with the control group (Figures 5A,B). As expected, we did not observe any significant change in GRP 78, eIF-2α or PERK in the group that underwent single sevoflurane exposure (Supplemental Figures S1C,D). These results suggest that the activation of PERK pathway may promote ER stress as a consequence of repeated but not single sevoflurane exposure.

### TUDCA reversed the decrease in the expression of synaptic-plasticity-associated proteins in the sevoflurane-exposed hippocampus

**Figure 6:**
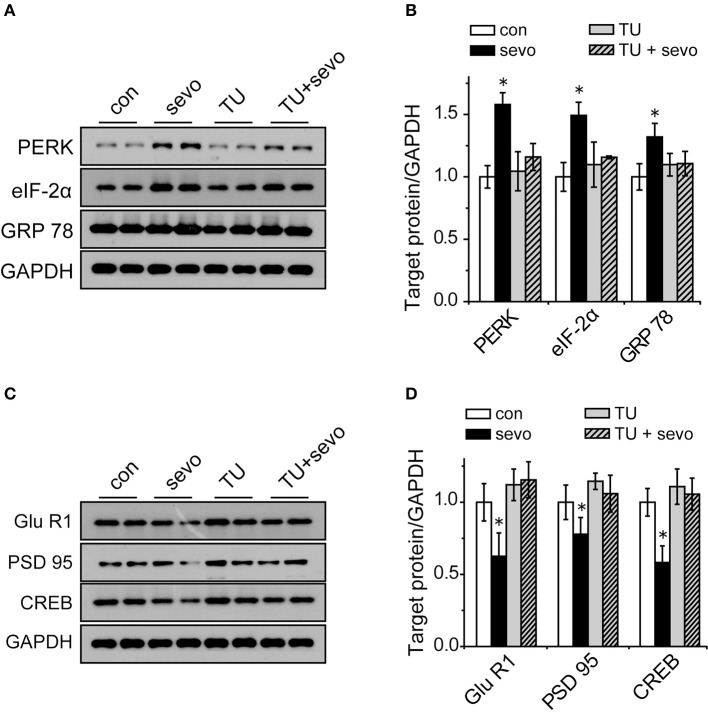
Increased expression of GRP 78, PERK, and eIF-2α and decreased expression of GluR1, PSD95, and CREB were reversed by the ER stress inhibitor TUDCA. **(A)** Representative samples showing the expression levels of GRP 78, PERK and eIF-2α in the hippocampi of the control group, the sevoflurane group, the TUDCA group and the TUDCA+sevoflurane group; **(B)** Densitometric analysis of GRP 78, PERK, and eIF-2α. *N* = 3–6 times for each protein. One-way ANOVA; ^*^p < 0.05 vs. control. Data are presented as the means ± SEM. **(C)** Representative samples showing the expression levels of GluR1, PSD95, and CREB in the hippocampi of the control group, the sevoflurane group, the TUDCA group and the TUDCA+sevoflurane group; **(D)** Densitometric analysis of GluR1, PSD95, and CREB. One-way ANOVA; ^*^p < 0.05 vs. control. Data are presented as the means ± SEM.

To clarify the correlation between ER stress and synaptic plasticity, we injected the rats with TUDCA (an ER stress inhibitor) intraperitoneally before each sevoflurane exposure, and then we analyzed the levels of ER-stress-associated proteins and synaptic-plasticity-related proteins in the hippocampus. The results showed that the expression levels of GRP 78, PERK and eIF-2α were significantly increased in the sevo group and that the increases were reversed by application of TUDCA (Figures 6A,B). The expression levels of GluR1, PSD95, and CREB were significantly decreased in the sevo group, but the decrease in expression was prevented by treatment with TUDCA (Figures 6C,D). These results suggested that the ER stress inhibitor TUDCA, could reverse the changes in synaptic plasticity protein levels that were induced by repeated sevoflurane exposure in early life.

## Discussion

Our results showed that repeated short-term exposure to sevoflurane resulted in impairment of learning and memory. Furthermore, we found that proteins related to ER stress were unusually abundant and synaptogenesis-related proteins markedly decreased after repeated sevoflurane exposure, indicating that ER stress and dysfunction in hippocampal synaptic plasticity may be associated with deficits in spatial learning and memory induced by repeated sevoflurane exposure.

Most studies on the effects of sevoflurane have focused on a single prolonged exposure, whereas only a few studies have examined repeated short-term exposure. Extremely long exposure to sevoflurane is rare for infants in the clinic, whereas repeated short-term exposure to sevoflurane, although more common and well worth exploring, receives less attention.

In clinical studies, Flick et al. have found that repeated exposure to anesthesia before the age of 2 is a significant independent risk factor for the later development of learning disabilities (6); similarly, Wilder et al. have found that multiple exposure to anesthesia, but not single exposure, is a significant risk factor for the later development of learning disabilities in children (18). Our results indicated that both learning and memory were impaired by repeated exposure to sevoflurane, in accordance with those clinical studies.

Synaptic plasticity is closely related to the structural modification of synapses and to synaptic transmission (19). The impairment of synaptic plasticity can lead to deficits in cognitive function (8). LTP is widely considered the neuronal basis for learning and memory. A change in LTP function can be reflected in the strength of postsynaptic neurotransmission. Our current results showed that repeated sevoflurane exposure markedly impaired LTP at CA3/Schaffer collateral-CA1 synapses of the hippocampus, in agreement with findings from previous studies (20). Furthermore, the inhibition of LTP caused weakness in postsynaptic transmission and changes in the morphological and structural development of synapses.

The reduction of LTP may be related to the expression of synaptic proteins. We found that the expression levels of GluR1, PSD95 and CREB in the hippocampus were all markedly decreased in rats that received repeated sevoflurane exposure. GluR1 is a glutamate receptor and cation channel that is integral to plasticity and synaptic transmission at many postsynaptic membranes. The underlying physiological correlate of increases in EPSP size is postsynaptic upregulation of GluR1 at the membrane (21). The upregulation of GluR1 at the membrane results in a long-lasting increase in EPSP size, which underlies LTP. In the current study, repeated sevoflurane exposure downregulated the expression of GluR1 and led to reduced LTP. The transcription of GluR1 in long-term memory is controlled through CREB. CREB is a cellular transcription factor that has been extensively linked to learning, memory (22) and LTP (23). Gene transcription of the transcription factor CREB is closely related to hippocampus-mediated memory consolidation (24). In agreement with results from previous studies (25–27), our western blotting results revealed that repeated sevoflurane exposure was associated with downregulated expression of CREB, which led to a decrease in neurogenesis (28). PSD95 is a postsynaptic marker (29). Downregulated expression of PSD95 results in disrupted synaptic structure (30, 31). The decrease in synaptogenesis-related proteins in the hippocampus indicated deficits in synaptic plasticity, which might lead to dysfunction of spatial learning and memory.

Previous studies have revealed that sevoflurane induces Ca^2+^ release from the ER to the cytosol via the inositol 1,4,5-trisphosphate receptor (IP3R) and ryanodine receptor (RyR), which suggests an influence of sevoflurane on calcium homeostasis in neurons (32–34). However, whether repeated short-term sevoflurane exposure can induce ER stress that impairs synaptic plasticity and long-term memory is still unknown. In our study, repeated sevoflurane exposure increased the expression of GRP 78, PERK and eIF-2α. GRP 78 is a crucial biomarker of ER stress (15). When misfolded proteins accumulate, GRP 78 is released, thereby permitting aggregation of transmembrane signaling proteins and triggering the ER stress response (15). PERK is a critical transmembrane signaling protein (35), and eIF-2α is a key protein in the PERK pathway (36). TUDCA is a neuroprotective drug via inhibiting ER stress (37). We found that TUDCA reversed the decline in the expression of GluR1, PSD95, and CREB. These results revealed that ER stress might play a critical role in deficits of synaptic plasticity, which may correspond to the recent finding that impaired LTP could be rescued by the ER stress inhibitor TUDCA in a chronic intermittent hypoxia model (14).

## Conclusions

Our results showed that repeated short-term exposure to sevoflurane resulted in impairment of learning and memory abilities, and the underlying mechanism included hippocampal synaptic dysfunction and ER stress. ER stress and decreased expression of synaptic proteins may be involved in this impairment of synaptic function, which can be reversed by the ER stress inhibitor TUDCA.

## Author contributions

YL and Z-QL designed and supervised the project. F-YS, Y-CS, FG, and Z-DX performed behavioral test and western-blotting experiments. FG, BZ, and Y-QM performed electrophysiological experiments. F-YS, Y-QZ, RL, and BZ performed ER stress experiments. F-YS, BZ, Z-DX, YL, and Z-QL wrote the manuscript. All authors discussed the data and read the manuscript.

### Conflict of interest statement

The authors declare that the research was conducted in the absence of any commercial or financial relationships that could be construed as a potential conflict of interest.
