# Supplementary material for: Cognitive Impairment and Endoplasmic Reticulum Stress Induced by Repeated Short-Term Sevoflurane Exposure in Early Life of Rats
Source: Front Psychiatry. 2018 Aug 2;9:332. doi: 10.3389/fpsyt.2018.00332 (PMC6083612; doi:10.3389/fpsyt.2018.00332)
Supplement: Supplementary file 1 [file Data_Sheet_1.pdf]

Supplementary Information

Cognitive Impairment and Endoplasmic Reticulum Stress Induced by Repeated Short-Term Sevoflurane Exposure in Early Life of Rats

Fu-Yi Shen[1#], Ying-Cai Song[1#], Fei Guo[2#], Zhen-Dong Xu[1], Qian Li[1], Bing Zhang[1,2,3], Yuqin Ma[2,3], Yue-Qi Zhang[1], Rong Lin[1], Yang Li[2*], Zhi-Qiang Liu[1*]

Supplemental methods

Animals

Four-day-old new-born Sprague Dawley (SD) male rats obtained from Shanghai Sippr-BK Laboratory Animal Co were housed with their dams under controlled illumination (12-h/12-h light/dark cycle, light from 07:00 to 19:00) at 24±1 °C. The rats were given free access to food and water. On postnatal day (P) 7, all neonatal rats were randomly divided into 2 groups: a control group and a sevoflurane (sevo) group. The neonatal rats in the sevo group were exposed to 3% sevoflurane for 2 h on P7, whereas those in the control group were subjected to the same conditions without receiving sevoflurane. Changes in synaptic plasticity and ER stress-associated protein levels were then analysed by western blotting (n=4 for each group).

Anaesthesia

The neonatal rats in the sevo group received 3% sevoflurane in 50% O_2/N_2 for 2 h on P7 in a sealed box at a set temperature of 37±1°C. The rats in the control group received 50% O_2/N_2 at the same flow rate in a similar box on the same day. The neonatal rats breathed spontaneously. The concentrations of sevoflurane and oxygen were measured continuously (Vamos, Drager, Germany). After sevoflurane exposure, the neonatal rats were returned to their dams after recovering their righting reflex.

Western-blotting analysis

Six hours after sevoflurane exposure completed (P7), the rats were deeply anaesthetized with an injection of chloral hydrate (400 mg/kg, intraperitoneally). Immediately after decapitation, the hippocampus was dissected and homogenized by sonic disruption. The homogenate was centrifuged at 14,000 rpm for 10 min at 4°C. Total protein concentrations were measured using

a BCA assay (Pierce, Rockford, IL). The proteins were separated on SDS-PAGE gels and transferred to nitrocellulose membranes. The membranes were blocked and then incubated overnight at 4℃ with primary antibodies (anti-GRP 78, 1:1000, Sigma-Aldrich; anti-PERK, 1:1000, Sigma-Aldrich; anti-eIF-2α, 1:1000, Sigma-Aldrich; anti-phospho-AMPA receptor, GluR1 subunit (pSer845), 1:1000, Sigma-Aldrich; anti-PSD95, 1:1000, Sigma-Aldrich; anti-CREB, 1:1000, Sigma-Aldrich; anti-GAPDH, 1:1000, Sigma-Aldrich). Band intensities were measured using Image Processing and Analysis in Java (ImageJ) software.

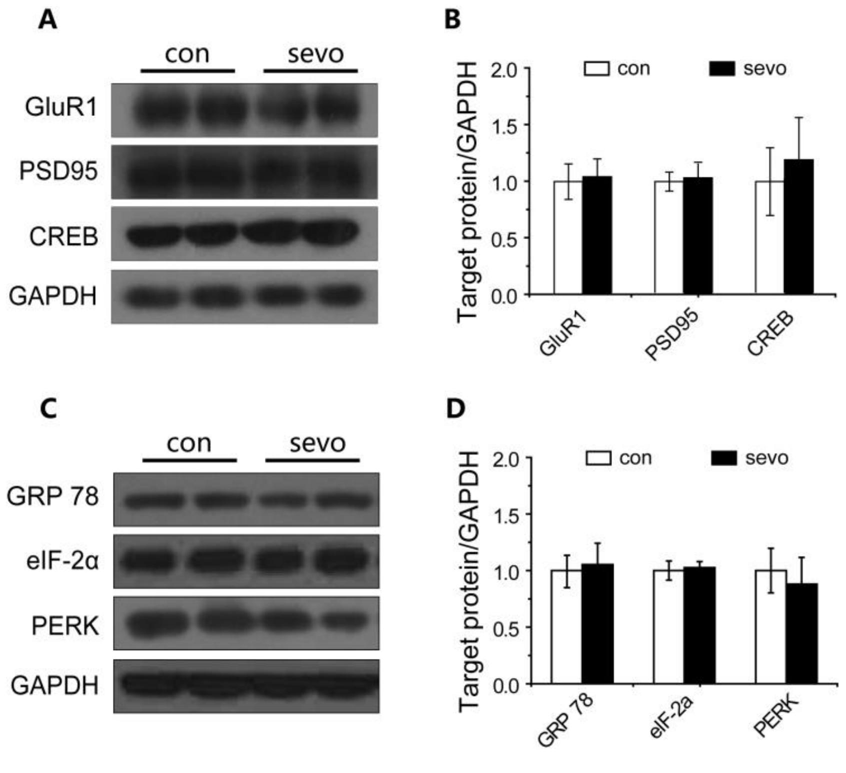

Supplement Figure 1. Single sevoflurane exposure has no effect on the expression of synapse protein and ER stress. (A, C) Representative samples showing the expression levels of GluR1, PSD95, CREB, GRP 78, PERK and eIF-2α in the hippocampus of the control group and the sevoflurane group; **(B, D).** Densitometric analysis of GluR1, PSD95, CREB, GRP 78, PERK and eIF-2α. N=4-6 times for each protein.

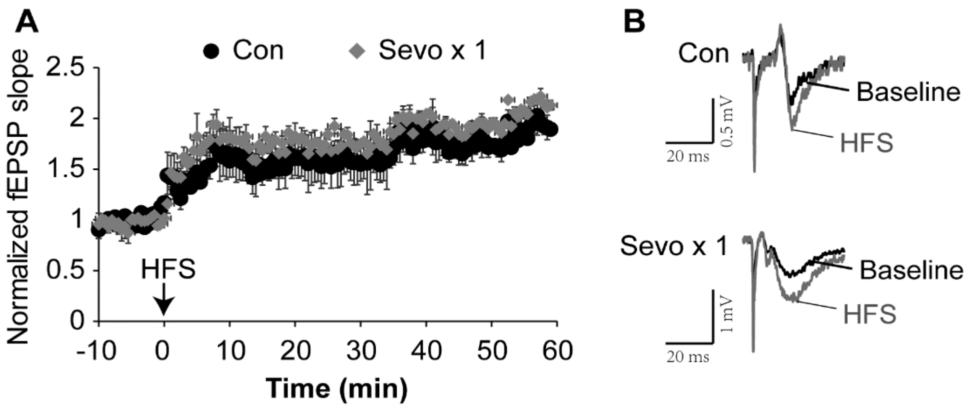

Supplement Figure 2: single sevoflurane exposure didn't neutralized LTP in the hippocampus. A. Tetanic stimulation (100 Hz for one time) induced LTP in the Schaffer collateral-CA1 pathway of rat hippocampal slices. B. Representative traces of the fEPSP waves were recorded at baseline (the black line) and after repeated sevoflurane exposure (the grey line). Early-life single sevoflurane exposure didn't impaired LTP in the rat hippocampus. N=6 slices, 3 rats for control group; n=7 slices, 3 rats for sevo group.
